# A Case of Hysteric Trance

**Published:** 1886-12

**Authors:** C. F. Darnall

**Affiliations:** West Union, Iowa


### A REPORT OF A CASE OF HYSTERIC TRANCE. *By* C. F. DARNALL, M.D.

Mrs. H. A. H., aged 31, came under the writer's care in September, 1884. She had been married fifteen years, and had given birth to nine children, the youngest being at that time sixteen months old. The patient was a farmer's wife, a household drudge, small and poorly developed, of a phthisical family, nervous temperament, and of ordinary mental capacity. For years she had been afflicted with cough and expectoration and a gradual failure of physical forces. A sudden and severe cold caused a pleurisy, which was the occasion for calling medical assistance. After an illness of a few days and a complication of ailments she convalesced, but an irritable stomach and aggravating cough remained. In the fourth week she was able to be out of bed a short time during each day, when the climax came in the shape of a quarrel with her husband. She had menstruated a few days before, and at this time was seized with an acute pain in the left ovarian region. A few hours after, when conversing about her marital troubles, she suddenly went into a trance. The first thing noticed was a vacant stare and a catching of the breath, a throwing out of the arms at right angles to the body, her legs extended and head thrown back, and then she became unconscious. Her limbs were stiff and resisted all attempts to unbend; the face was pallid, eyelids tightly closed and closing again if forced open; eyes turned upward and moving when touched; pupils slightly dilated and slowly responding to the light. The respiration was shallow and irregular, seven to nine per minute; pulse, 78 to 90, soft and weak; flatness on percussion, sibilant and subcrepitant *râles.* Superficial temperature was decreased, generally 98° in the axilla; sometimes normal, and rarely 99° or 100°, which was the case when the attack was brought on by any high nervous excitement. Liquid food was slowly swallowed, often being retained for quite a while before causing sufficient irritation to relax the pharyngeal muscles. These variations embrace the symptoms of many attacks she had subsequently. For the first week or so they would come on at eight or half past in the morning and at six in the afternoon, and lasting from one to three hours. The aura would be pain in the stomach, shifting to the left ovary, a slight chill, then the trance. After the initial rigidity passed away, in half an hour or so, she would still be stiff, but the limbs could be moved, the other conditions being the same as at the beginning. Muscular relaxation would all at once take place and she would waken with a start. She seemed then to not understand what had happened; would be restless and change her position in bed; would not speak, but motion for her limbs to be rubbed, for a drink of water, or some such thing. Then she would fall into a natural, deep sleep, lasting from a few moments to a half hour, from which she would awaken with a clear mind, complaining of a general feeling of soreness and fatigue. In the sixth week family matters reached a crisis, her husband leaving her, but returning occasionally to talk over the division of the property. At this time her attacks lost all periodicity, coming oftener and at any time, with less force and of shorter length. If she happened to be sitting in a chair when overcome, she would retain the erect position. For a time there was each day a series of them, each one shorter and weaker than the one preceding, with a period of sleep intervening, appearing as if her nervous force had assumed a tangible form, wave succeeding wave until lost in quietude again.

Her friends then unadvisedly called in a minister to look after her spiritual necessities, and at the next trance, a little while after, a surprising change took place. Coming in between the stage of lethargic rigidity and the sleep was an apparently lucid interval, during which she would sit up in bed. The eyes were opened wide, filled with a far-away supernatural look, and her expression of countenance was that of one engaged in delightful, dreamy contemplation. It seemed as if another spirit had taken momentary possession of her body, revealing entirely different views and impulses from those which she exhibited when surrounded by ordinary every-day environments. Before, her life had not been governed by religious motives, but now the whole bent of her mind was directed in elevated and exalted channels. Without the statuesque appearance of ecstacy, or the waxen mobility of catalepsy, she assumed a devotional attitude, lasting only a moment. At the visit of the minister a hymn book was left on the bed, and after she emerged from the trance state she made search for it and opened it, when through with the supposed prayer. She turned over leaf by leaf until she found an old familiar hymn known to her in bygone days. The words were unaccompanied by notes, yet she correctly carried the tune through and would sing one or two more. This was done several times a day and for about a week. Her voice was clear and sweet, vibrating in the most touching way. If the key was pitched too high or too low, she would begin again until she was right. If she mispronounced a word or sounded a wrong note, the word or note would be correctly repeated. If any one were to purposely place the book upside down or turn over too many leaves, she would right the book and proceed to the finish without being disconcerted. All this time she appeared awake, her eyes wide open, pupils dilated, deeply intent upon what she was doing and taking no note whatever of her friends or surroundings. This would last a short time, when she would close the book and sleep, awakening perfectly rational; the wandering spirit had departed its host, her own had returned, and she was herself again. No knowledge existed of what had transpired from the moment she entered the trance nor any memory of visions. All was blank and the time consumed was as nothing. Sometimes the book would be hidden or another substituted before she searched for it. If she could not find it she would look at her hands a few moments in a dazed, bewildered manner, shed a few hysterical tears and go to sleep.

Thus ran the case over three months of time, gradually submitting to treatments, as her domestic troubles were amicably adjusted. Nothing was done to shorten or avert the attacks, yet it looked as if Charcot's method of ovarian compression always relieved her, as she would return to rationality whenever it was employed. As to physical condition, there was enlargement of the left ovary and thickening of the left broad ligament; uterus in normal position, enlarged somewhat, with endometritis and granular erosions. Hysteria disappeared when these local troubles were cured, but owing to the complexities of the case and the many discouraging features arising, recovery was necessarily delayed. Menstruation occurred every two or three weeks; and while she was in bed nearly all the time, yet hardly a day passed that she did not take a *secret smoke*, knowing it was forbidden.

Many remedies were tried, but the bromides, tonics and good food were successful. The monthly flow became normal, and for two years now there have been only once hysteric symptoms, and then it was *globus hystericus* in the throat and dyspnœa, controlled by the bromides.

Sufficient space has already been taken without entering into speculation as to the pathology of the case, and our text-books will abundantly supply that.

West Union, Iowa.

